# Photosynthetic Pigments and Biochemical Response of Zucchini (*Cucurbita pepo* L.) to Plant-Derived Extracts, Microbial, and Potassium Silicate as Biostimulants Under Greenhouse Conditions

**DOI:** 10.3389/fpls.2022.879545

**Published:** 2022-05-18

**Authors:** Doaa Y. Abd-Elkader, Abeer A. Mohamed, Mostafa N. Feleafel, Asma A. Al-Huqail, Mohamed Z. M. Salem, Hayssam M. Ali, Hanaa S. Hassan

**Affiliations:** ^1^Department of Vegetable, Faculty of Agriculture (EL-Shatby), Alexandria University, Alexandria, Egypt; ^2^Plant Pathology Institute, Agriculture Research Center (ARC), Alexandria, Egypt; ^3^Chair of Climate Change, Environmental Development and Vegetation Cover, Department of Botany and Microbiology, College of Science, King Saud University, Riyadh, Saudi Arabia; ^4^Forestry and Wood Technology Department, Faculty of Agriculture (El-Shatby), Alexandria University, Alexandria, Egypt

**Keywords:** zucchini plants, beneficial microbes, plant-derived biostimulants, potassium silicate, phenolic compounds

## Abstract

There are many technological innovations in the field of agriculture to improve the sustainability of farmed products by reducing the chemicals used. Uses of biostimulants such as plant extracts or microorganisms are a promising process that increases plant growth and the efficient use of available soil resources. To determine the effects of some biostimulants' treatments on the photosynthetic pigments and biochemicals composition of zucchini plants, two experiments were conducted in 2019 and 2020 under greenhouse conditions. In this work, the effects of beneficial microbes (*Trichoderma viride* and *Pseudomonas fluorescens*), as well as three extracts from *Eucalyptus camaldulensis* leaf extract (LE), *Citrus sinensis* LE, and *Ficus benghalensis* fruit extract (FE) with potassium silicate (K_2_SiO_3_) on productivity and biochemical composition of zucchini fruits, were assessed as biostimulants. The results showed that *E. camaldulensis* LE (4,000 mg/L) + K_2_SiO_3_ (500 mg/L) and *T. viride* (10^6^ spore/ml) + K_2_SiO_3_ (500 mg/L) gave the highest significance yield of zucchini fruits. Furthermore, the total reading response of chlorophylls and carotenoids was significantly affected by biostimulants' treatments. The combination of K_2_SiO_3_ with *E. camaldulensis* LE increased the DPPH scavenging activity and the total phenolic content of zucchini fruits, in both experiments. However, the spraying with K_2_SiO_3_ did not observe any effects on the total flavonoid content of zucchini fruits. Several phenolic compounds were identified *via* high-performance liquid chromatography (HPLC) from the methanol extracts of zucchini fruits such as syringic acid, eugenol, caffeic acid, pyrogallol, gallic acid, ascorbic acid, ferulic acid, α-tocopherol, and ellagic acid. The main elemental content (C and O) analyzed *via* energy-dispersive X-ray spectroscopy (EDX) of leaves was affected by the application of biostimulants. The success of this work could lead to the development of cheap and easily available safe biostimulants for enhancing the productivity and biochemical of zucchini plants.

## Introduction

In agricultural performance, plant biostimulants are including different bioactive natural substances such as plant extracts, beneficial microorganisms, macroalgae seaweeds extracts, humic acid, fulvic acid, silicon, animal protein hydrolysate, vegetal protein hydrolysate, and bacteria belonging to the genera *Azotobacter, Rhizobium*, and *Azospirillum* (Chiaiese et al., 2018; Ricci et al., 2019).

In the recent years, the use of external preparations capable of stimulating plant growth by working on plant metabolism has become suitable for enhancing the efficiency of chemical fertilizers (Baroccio et al., 2017). Increasing yield is often associated with better-quality vegetables or fruits. According to the previous studies, biostimulants have a positive effect on the production of vegetables and fruits (Kocira et al., 2017; Goñi et al., 2018; Milić et al., 2018; Tarantino et al., 2018). In modern agriculture, biostimulants are the important strategies in the production of horticultural crops and consist of highly heterogeneous classes of compounds with a wide range of actions to improve quantitative and qualitative crops (Drobek et al., 2019).

Zucchini or squash (*Cucurbita pepo* L.), a highly polymorphic vegetable crop, is growing during the summer season in Egypt and all over the world (Ezzo et al., 2012; Mahmoud, 2016; Contreras et al., 2020), as a result of its economic importance and nutritional value. However, the increasing demand of consumers, in the local and international markets for fresh fruits of zucchini all year round, led to an increase in planting zucchini in the greenhouse (Formisano et al., 2020a,b). Zucchini is one of the most significant vegetable cash crops, especially, in newly reclaimed areas of Egypt, due to its high-yielding potential per unit area in the short-growing season. Therefore, improving the agricultural practices of zucchini production is of great economic interest. This may be achieved by applying simple applicable modern and low-cost strategies such as the use of silica compounds, *Trichoderma*, or plant-growth-promoting- rhizobacteria (PGPR) and plant extracts that stimulate the growth and development of this plant and then increase the productivity, which is safe for humans and environments (Savvas et al., 2009; Formisano et al., 2021; Novello et al., 2021).

Silicon (Si) is a biostimulant in the group of inorganic products. Foliar application of Si, as potassium silicates, is a relatively new technique of feeding vegetable plants, with several roles in plant physiology, regulation of ions uptake and increased tolerance of plants to various biotic and abiotic stresses (Artyszak, 2018). Moreover, Si stimulates the growth, development, and yield components of many vegetable species by correcting the levels of endogenous growth hormones (Artyszak, 2018).

Inoculating vegetable plants with *Trichoderma* or PGPR may be an effective strategy to stimulate the growth and development of plants as well as to minimize the use of synthetic fertilizers and agrochemicals. This strategy can improve plant tolerance for the abiotic stresses through induction of resistance by the production of phytohormones, enhancing soil productivity and volatile compounds that affect the plant signaling pathways (Kumar et al., 2020; Mannino et al., 2020). *Trichoderma* spp. are free-living filamentous fungi in the soil, and some of them are the most potent agents for the biocontrol of soil-borne plant pathogens (Castiglione et al., 2021). *Trichoderma* can improve soil nutrient availability and promote plant growth and biostimulant (Velmourougane et al., 2019; Chen et al., 2021).

Plant extracts contain many bioactive compounds such as sugars, amino acids, proteins, nucleic acids, polysaccharides (Fernie and Pichersky, 2015), phenolic acids, and flavonoids (Sarker and Oba, 2018; Salem et al., 2021b). Foliar application of plant extracts leads to stimulating the root growth, photosynthetic capacity, and increasing the nutrient use efficiency, which ultimately leads to the growth promotion of vegetable crops (Bulgari et al., 2015). In addition, phenolic acids and flavonoids often play the important roles in the plant's defense against disease (Sarker and Oba, 2018). However, the plant extracts can be considered a good source of natural antioxidants and antimicrobial in both *in vitro* and *in vivo* (Di Mola et al., 2019; Souri and Bakhtiarizade, 2019; Godlewska et al., 2021). The raw materials resulting from the pruning processes of *Eucalyptus camaldulensis, Citrus sinensis*, and *Ficus benghalensis* trees are readily available in high quantities in Egypt. The growth of these trees under Egyptian conditions is very suitable and therefore economical in use. Further, the extracts from these trees were shown potential activities against the growth of bacteria and fungi (Nair and Chanda, 2007; Ekwenye and Edeha, 2010; El-Hefny et al., 2017; Bhawana et al., 2018; Salem et al., 2019; Abdelkhalek et al., 2020; Abo-Elgat et al., 2020; Afzal et al., 2020; Fatima et al., 2020).

The present research was carried out as an attempt to apply simple applicable modern, low-cost, and safe strategies through studying the effect of the use of some natural biostimulant with silicon on the productivity and bioactive component responses of zucchini plants, grown in clay soil, under drip irrigation in the greenhouse.

## Materials and Methods

A total of two consecutive experiments were carried out in the years 2019 and 2020 under a drip irrigation system in the greenhouse, at the Experimental Station Farm of the Faculty of Agriculture, Alexandria University, Abies, Alexandria, situated in Egypt, 31° 13′ N latitude, 29° 59′ E longitude.

### Soil Analysis

Prior to the initial of the first experiment, soil samples of the experimental site up to 30 cm depth were collected and analyzed for some chemical and physical properties according to the standard procedures (Sparks et al., 2020). The main physical and chemical soil characteristics at the experimental site with clay soil were 46% sand, 24% silt, and 30% clay, electrical conductivity (EC): 2.60 dS m^−1^, pH: 8, total nitrogen (N): 0.16%, phosphorus and potassium were 0.30 ppm and 0.33 m eq l^−1^, respectively.

### Experimental Work and Treatments

The current experiments were performed to study the effect of plant extracts, K_2_SiO_3_, and microbial inoculation with *Trichoderma viride* (10^6^ spore/ml) and *Pseudomonas fluorescens* (10^8^ CFU/ml) as biostimulants on the growth, productivity, and bioactive of zucchini fruit that is grown in greenhouse conditions. Extracts were prepared from *Eucalyptus camaldulensis* leaves, *Citrus sinensis* leaves, and *Ficus benghalensis* fruits at the concentration of 4,000 mg/L as recommended in our previous work (Hassan et al., 2021).

AZIAD F1 cultivar was used in this study, and this cultivar was imported from the Sakata Tacky Company Japan vegetable seed. It is a desirable variety in the Egyptian market and bears low temperatures and high production (Abd Elmohsen et al., 2021). Zucchini seeds were sown in late September and in late December, in the first and second experiments, respectively. Within the same plantation row, the spacing between each plant and the other was set to be 30 cm, whereas the spacing between each line and the other was 1 m (3 plants/m^2^). The experimental layout was a randomized complete block design (RCBD), with three replicates. RCBD is used to control the variation in the experiment by accounting for spatial effects in the field or greenhouse, e.g., the variation in soil fertility or drainage differences in the field (Lauren, 2014). Each trial consisted of 13 treatments as shown in Table 1.

**Table 1:** Treatments used in this study.

**Treatment**	**Combination of biostimulants**
Control	Without any microbial, plant extract, and potassium silicate
*T. viride*	*Trichoderma viride (*10^6^ spore/ml)
*T. viride* + K_2_SiO_3_	*T. viride* (10^6^ spore/ml) + potassium silicate (500 mg/L)
*P. fluorescens*	*Pseudomonas fluorescens* (10^8^ CFU/ml)
*P. fluorescens* + K_2_SiO_3_	*P. fluorescens* (10^8^ CFU/ml) + potassium silicate (500 mg/L)
*T. viride* + *P. fluorescens*	*T. viride + P. fluorescens*, individually (10^6^ spore/ml +10^8^ CFU/ml)
*T.viride* + *P. fluorescens* + K_2_SiO_3_	*T. viride + P. fluorescens*, individually (10^6^ spore /ml +10^8^ CFU/ml) + potassium silicate (500 mg/L)
*E. camaldulensis* LE	*Eucalyptus camaldulensis* leaf extract (4,000 mg/L)
*E.camaldulensis* LE + K_2_SiO_3_	*E. camaldulensis* leaf extract (4,000 mg/L) + potassium silicate (500 mg/L)
*C. sinensis* LE	*Citrus sinensis* leaf extract (4,000 mg/L)
*C. sinensis* LE + K_2_SiO_3_	*C. sinensis* leaf extract (4,000 mg/L) + potassium silicate (500 mg/L)
*F. benghalensis* FE	*Ficus benghalensis* fruit extract (4,000 mg/L)
*F.benghalensis* FE + K_2_SiO_3_	*F. benghalensis* fruit extract (4,000 mg/L) + potassium silicate (500 mg/L)

Each treatment was replicated three times, and each replicate consisted of 35 plants. Water irrigation was applied through the drip irrigation system. The drip irrigation system consisted of laterals GR of 16 mm in diameter with emitters at a 0.3-m distance. The emitters had a discharge rate of 4 L/h. The actual evapotranspiration of the zucchini crop (ETc), under greenhouse at “Abies, Alexandria” area conditions, was calculated and adjusted at the start of each growth stage (Feleafel and Mirdad, 2014). It was calculated by multiplying reference evapotranspiration (ET_0_) for different growth stages through both two experiments (from late September 2019 up to mid-December, 2019, in the first experiment and from late December and ended in late February 2020, in the second experiment) by a crop coefficient (K_C_); ET_c_ = ET_0_ × K_C_ (Allen, 1998; Razmi and Ghaemi, 2011) as shown in Supplementary Table S1. Irrigation frequency was every 5 days, to maintain soil water above 50% soil water depletion (Qassim and Ashcroft, 2002), which is the optimum level for zucchini plants.

**Figure 1:**
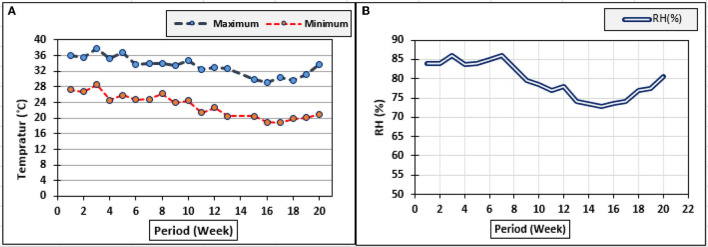
The measured climatic conditions in the greenhouse during 2019 and 2020. **(A)** Minimum and maximum temperature (°C); **(B)** RH (%).

The treatments of inoculation with *T. viride* were used as a drench to the plants' root area, were done through the addition of 50 ml suspension, which was mixed thoroughly with the soil, and then watered and left to ensure establishment and distribution in the soil. However, inoculation with *P. fluorescens, T. viride* + *P. fluorescens* and application of K_2_SiO_3_ and three extracts from *E. camaldulensis* LE, *C. sinensis* LE, and *F. benghalensis* FE were sprayed separately on zucchini plants. All treatments were added to the plants four times during the entire growing season of zucchini plants. The first addition was after 2 weeks from the sowing date, and then, the addition was done weekly. The spraying was done for each biostimulant separately, and *P. fluorescens* was sprayed before the spraying with K_2_SiO_3_ at an interval of 3 days. Ammonium sulfate (NH_4_)_2_SO_4_, (20.5% N), phosphoric acid (58%), and potassium sulfate (48% K_2_O) were the sources of N, P_2_O_5_, and K_2_O, respectively. Nitrogen, phosphorus, and potassium fertilizers were fertigated at rates of 143, 167, and 238 kg N, P_2_O_5_, and K_2_O/ha, respectively, which were injected directly into the irrigation water (fertigation) using a venture injector at one time weekly through a drip irrigation system in equal doses, starting from 2 weeks after planting. Climatic data, such as maximum and minimum air temperature (T_maximum_ and T_minimum_ °C) and relative humidity (RH%), were collected using Testo 175-H1 as shown in Figure 1.

### Evaluation of Productivity

Zucchini fruits were harvested after 50 days from sowing, at a rate of two times a week. For each harvest, the number of fruits was counted and weighed for each treatment and then attributed to the hectare. The number and weight of fruits were recorded after each harvesting accumulatively. The number of fruits/hectare and the total productivity/hectare for each treatment were calculated.

### Photosynthetic Pigments

Extraction of chlorophylls (a and b) and carotenoids from zucchini leaves was performed in ethanol 96% (v/v) in a proportion of 1:10 (w/v). The absorbance was read at 664 and 649 nm for chlorophylls a and b, respectively, as well as at 470 nm for carotenoids (Lichtenthaler, 1987; Campobenedetto et al., 2021).

### Energy-Dispersive X-Ray Spectroscopy (EDX) Analysis

Energy-dispersive X-ray spectroscopy analysis was performed to measure the changes in the elemental chemical composition of zucchini leaves (nine leaves for each treatment) due to different treatments with treated with 500 mg/L K_2_SiO_3_ and control treatment. Energy-dispersive spectrometry (EDX), JFC-1100E ion sputtering device (model JEOL/MP, JSMIT200 Series, Tokyo, Japan) with acceleration voltage of 20.00 kV to show the elemental compotation at three point was used (Salem et al., 2021a).

### 2,2-Diphenyl-1-Picrylhydrazyl (DPPH) Radical Scavenging Activity

At 1 ml of 0.1 mM 2,2-diphenyl-1-picrylhydrazyl (DPPH), we added different concentrations of the prepared zucchini fruits' methanolic extracts. After vigorous shaking, the mixture was incubated for 30 min in the dark and at 25°C. The reduction of the radical DPPH resulting from the incubation with the different dilutions of zucchini methanolic extracts was monitored by reading the color decrease at 517 nm (Mannino et al., 2020). The percentage DPPH radical scavenging activity was calculated using the formula: Inhibition (%) = [(A_control_ – A_sample_)/A_control_] × 100, where A_control_ and A_sample_ are the absorbance of the control and treatments (Abd-Elkader et al., 2021).

### Total Phenolic and Flavonoid Contents

Total phenolic content (TPC) was determined *via* Folin–Ciocalteu assay, as previously reported (Mannino et al., 2020). Quantification was performed using an external calibration curve with gallic acid (GA). Analyses were performed in triplicate, and data were expressed as millimole GA equivalents (GAE) per 100 g of FW., while the aluminum chloride colorimetric assay was used for total flavonoid content (TFC) determination and read at 510 nm using UV–Visible spectrophotometer Model UV 1601 version 2.40 (Shimadzu) (Marinova et al., 2005). Total flavonoids' content was expressed as mg catechin equivalents.

### Fruit Extraction and HPLC Analysis of Phenolic Compounds

The extraction process was carried out on zucchini fruit samples treated with plant extracts and microbial with 500 mg/L K_2_SiO_3_ and control treatment. A sample was taken for each treatment from the three replicates, about 15 fruits, then, all the fruits were grated and mixed well, and 30 g was taken then extracted by 60 ml methanol by the soaking method for 1 week (Ashmawy et al., 2020; Abd-Elkader et al., 2021). The extracts were then filtered through filter paper (Whatman no. 1) and then with a cotton plug. The extracts were concentrated and stored in brown vials in the refrigerator prior HPLC analysis. The phenolic compounds from the methanol extracts of each previous treatments were identified by the Agilent ChemStation [HPLC-(Agilent, Santa Clara, CA, USA)], which is composed of a quaternary pump and UV/Vis detector and C18 column (125 mm × 4.60 mm, 5 μm particle size). Chromatograms were obtained and analyzed using HPLC. Phenolic compounds were separated by employing a mobile gradient phase of water/acetonitrile/glacial acetic acid (980/20/5, v/v/v, pH 2.68) and acetonitrile/glacial acetic acid (1,000/5, v/v) with a flow rate of 1 ml/min and detected at 325 nm. All chemical standards (HPLC grade) were purchased from Sigma-Aldrich (St. Louis, MO, USA) (Hassan et al., 2021).

### Statistical Analysis

All data were analyzed by implementing the CoStat software version 6.303 (CoHort Software 798 Lighthouse Ave. PMB 320, Monterrey, CA, 93940, USA) package through a two-way analysis of variance (ANOVA). A Tukey's honestly significant difference (HSD) test (p < 0.05) was used to separate the means (Steel and Torrie, 1980).

## Results

### Productivity Parameters of Zucchini

**Figure 2:**
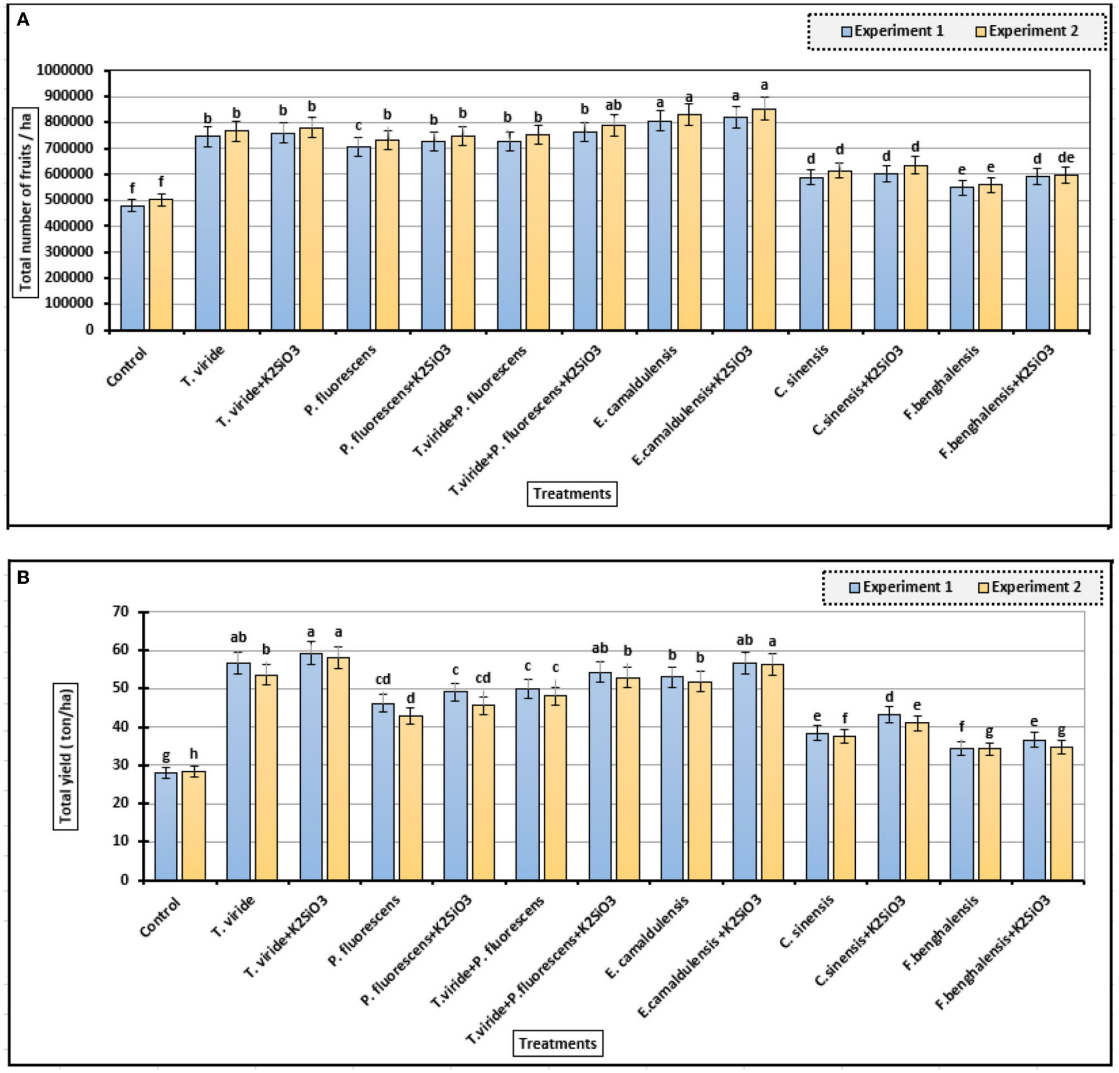
Fruits number and total yield of zucchini as affected by biostimulant treatments. **(A)** Total number of fruits/ha and **(B)** The total yield (ton/ha) (means ± S.E) of zucchini as affected by the extracts, microbial inoculations, and K_2_SiO_3_. Letters in the figure indicated that the means ± S.E of treatments with the same letter/s were not significantly different according to Tukey's HSD level of probability.

Figure 2A shows that spraying of plants with K_2_SiO_3_ and biostimulants led to a significant increase in fruits number (ha) compared to control. The fruits number/ha was significantly increased as zucchini plants were treated with *E. camaldulensis* LE and *E. camaldulensis* LE + K_2_SiO_3_ in the two consecutive experiments. In addition, plants sprayed with microbial and plant extracts were significantly increased the fruits number/ha as compared to the control treatment. Moreover, spraying plants with K_2_SiO_3_ at 500 mg/L with microbial or plant extracts were maximized the increase in fruits number/ha compared to biostimulant treatments without K_2_SiO_3_ in the two experiments.

The total yield/ha of zucchini fruits (Figure 2B) showed a significant difference among treatments with the highest values of 59.25 and 58 ton/ha, with the treatment of *T. viride* + K_2_SiO_3_, in the first and second experiments, respectively. *E. camaldulensis* LE + K_2_SiO_3_ treatment, also, gave a higher mean value in both experiments (56.67 and 56.34 ton/ha). It is noted that the use of K_2_SiO_3_ with microbial and plant extracts as biostimulants led to an increase in total productivity/ha of zucchini in both experiments.

### Photosynthetic Pigments

Table 2 presents the effect of K_2_SiO_3_ and plant extract or beneficial microbes as biostimulants on the content of photosynthetic pigments (total chlorophylls, chlorophylls a, b, and total carotenoids) of zucchini leaves. The results indicated a significant increase in the content of pigments as affected by the biostimulant treatments. It is clear from the results that the values obtained from the different photosynthetic pigments were affected by the different treatments of the biostimulant used. *T. viride* + K_2_SiO_3_, and *E camaldulensis* + K_2_SiO_3_ treatments increased all the photosynthetic pigment contents, but the effect was most significant by the treatment *E. camaldulensis* LE+ K_2_SiO_3_, in both experiments. In addition, Table 2 shows the application of the treatments *T. viride* and *E. camaldulensis* LE improved the chlorophyll pigment of leaves compared to the other treatments, in both experiments.

**Table 2:** Effect of plant extracts, microbial inoculations, and potassium silicates biostimulants, on photosynthetic pigments content of leaves in the second experiment.

**Treatment**	**Experiment 1**				**Experiment 2**			
	**Chlorophyll a**	**Chlorophyll b**	**Total chlorophylls**	**Total carotenoids**	**Chlorophyll a**	**Chlorophyll b**	**Total chlorophylls**	**Total carotenoids**
	**(mg/100 g fw)**	**(mg/100 g fw)**	**(mg/100 g fw)**	**(mg/100 g fw)**	**(mg/100 g fw)**	**(mg/100 g fw)**	**(mg/100 g fw)**	**(mg/100 g fw)**
Control	14.8 ± 0.21e	10.3 ± 0.13e	25.1 ± 0.51f	2.83 ± 0.32e	15.7 ± 0.33e	11.4 ± 0.55e	27.1 ± 0.13e	2.92 ± 0.13d
*T. viride*	24.6 ± 0.11ab	18.3 ± 0.25ab	42.9 ± 0.32ab	3.62 ± 0.42b	24.9 ± 0.24ab	18.8 ± 0.31ab	43.7 ± 0.21ab	3.72 ± 0.32ab
*T. viride* + K_2_SiO_3_	25.3 ± 0.12a	19.0 ± 0.14a	44.3 ± 0.43a	3.81 ± 0.11ab	25.6 ± 0.42a	19.7 ± 0.44a	45.3 ± 0.21a	3.97 ± 0.14a
*P. fluorescens*	20.1 ± 0.12bc	16.3 ± 0.52bc	36.4 ± 0.22cd	3.32 ± 0.14c	21.1 ± 0.11bc	16.9 ± 0.23b	38.0 ± 0.10c	3.48 ± 0.33ab
*P. fluorescens* + K_2_SiO_3_	21.2 ± 0.31b	17.0 ± 0.32b	38.2 ± 0.31c	3.49 ± 0.15b	22.0 ± 0.14bc	17.8 ± 0.21ab	39.8 ± 0.42bc	3.56 ± 0.42ab
*T. viride* + *P. fluorescens*	22.1 ± 0.13b	18.0 ± 0.22ab	40.1 ± 0.11b	3.45 ± 0.21ab	22.9 ± 0.51bc	17.9 ± 0.24ab	40.8 ± 0.14bc	3.50 ± 0.16b
*T. viride* + *P. fluorescens* + K_2_SiO_3_	23.4 ± 0.11ab	19.1 ± 0.11a	42.5 ± 0.10ab	3.59 ± 0.23b	24.0 ± 0.33ab	20.1 ± 0.25a	4.1 ± 0.23ab	3.64 ± 0.17ab
*E. camaldulensis*	24.4 ± 0.23ab	18.9 ± 0.21ab	43.3 ± 0.10ab	3.90 ± 0.22a	25.0 ± 0.22a	19.3 ± 0.51a	44.3 ± 0.31ab	4.02 ± 0.23a
*E. camaldulensis* + K_2_SiO_3_	25.6 ± 0.11a	20.2 ± 0.33a	45.8 ± 0.22a	4.01 ± 0.24a	26.0 ± 0.14a	20.8 ± 0.34a	46.8 ± 0.26a	4.13 ± 0.52a
*C. sinensis*	17.7 ± 0.14c	12.4 ± 0.51cd	30.1 ± 0.32e	3.01 ± 0.11b	17.9 ± 0.21cd	12.9 ± 0.42d	30.8 ± 0.31d	3.12 ± 0.22c
*C. sinensis* + K_2_SiO_3_	18.1 ± 0.21c	13.9 ± 0.22c	32.0 ± 0.12d	3.15 ± 0.32c	19.2 ± 0.41c	15.2 ± 0.32c	34.4 ± 0.42cd	3.21 ± 0.13bc
*F. benghalensis*	15.6 ± 0.05d	11.3 ± 0.11d	26.9 ± 0.33f	2.93 ± 0.36d	16.1 ± 0.44d	11.9 ± 0.52e	28.0 ± 0.34e	2.98 ± 0.16d
*F. benghalensis* + K_2_SiO_3_	17.3 ± 0.11c	14.9 ± 0.22c	32.2 ± 0.41d	3.10 ± 0.51c	18.2 ± 0.51c	14.2 ± 0.32c	32.4 ± 0.15cd	3.17 ± 0.05b

*Letters in the figure indicated that the means ± S.E of treatments with the same letter/within the same column were not significantly different according to Tukey's HSD at a 0.05 level of probability*.

### Elemental Compositions of Leaves by EDX Analysis

**Figure 3:**
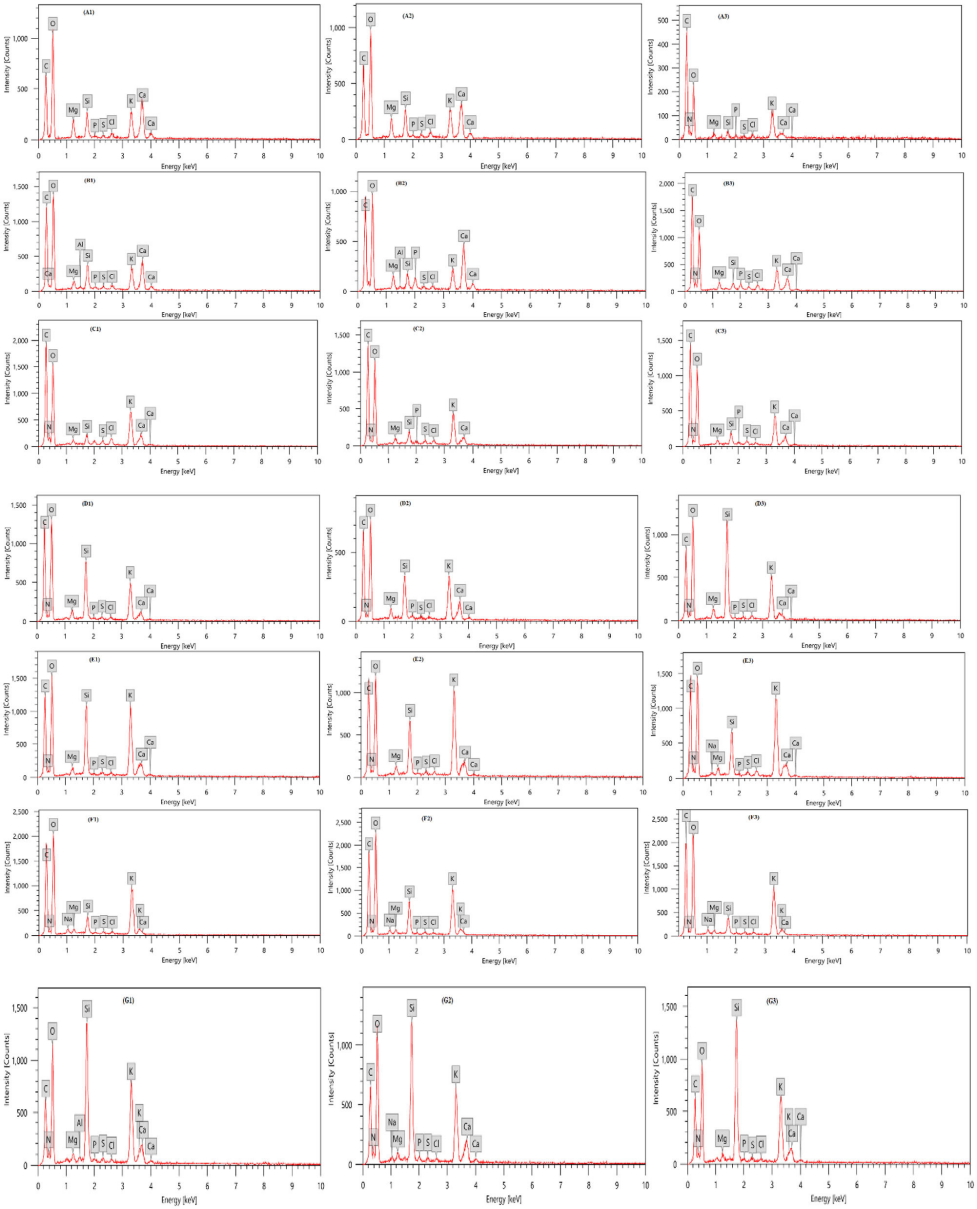
Elemental compositions of zucchini leaves with EDX analysis as affected by plant extracts, microbial inoculations, and potassium silicates biostimulants. Analysis was taken from three points for each treatment. **(A1–A3)** Control; **(B1–B3)** *T. viride* + K_2_SiO_3_; **(C1–C3)** *P. fluorescens* + K_2_SiO_3_; **(D1–D3)** *T. viride* + *P. fluorescens* + K_2_SiO_3_; **(E1–E3)** *E. camaldulensis* LE + K_2_SiO_3_; **(F1–F3)** *C. sinensis* LE + K_2_SiO_3_; **(G1–G3)** *F. benghalensis* FE + K_2_SiO_3_.

Table 3 and Figure 3 presents an EDX analysis to measure the changes in the elements' compositions of the leaves due to different treatments of biostimulants, which are six treatments in addition to the control. There was a significant effect of treatments on the elemental percentages with the highest values obtained with treatments *P. fluorescens* + K_2_SiO_3_, *T. viride* + *P. fluorescens* + K_2_SiO_3_, and *E. camaldulensis* LE + K_2_SiO_3_ on C, Mg, and Si elements. Also, there was a significant effect of treatments on elements N, P, and Ca percentages whereas the highest value was obtained by the plants treated with *T. viride* + K_2_SiO_3_. Moreover, the highest value of element K was obtained by treatment *E. camaldulensis* LE + K_2_SiO_3_.

**Table 3:** Effect of plant extracts, microbial inoculations, and potassium silicates biostimulants, on elemental composition (Atom%) of zucchini leaves in the second experiment.

**Treatment**	**Elements**									
	**C**	**N**	**O**	**Mg**	**Si**	**P**	**S**	**Cl**	**K**	**Ca**
Control	37.60 ± 0.33c	10.90 ± 0.88b	35.74 ± 0.93d	0.40 ± 0.05b	0.36 ± 0.04d	0.23 ± 0.03b	0.11 ± 0.01c	0.09 ± 0.01c	1.21 ± 0.04c	0.27 ± 0.04b
*T. viride* + K_2_SiO_3_	46.17 ± 0.22ab	25.19 ± 0.57a	40.21 ± 0.64c	0.16 ± 0.02d	0.77 ± 0.03c	0.64 ± 0.03a	0.12 ± 0.01b	0.15 ± 0.01ab	1.97 ± 0.08c	3.01 ± 0.06a
*P. fluorescens* + K_2_SiO_3_	47.88 ± 0.20a	4.28 ± 0.34c	43.65 ± 0.50b	0.39 ± 0.03c	0.63 ± 0.03c	0.13 ± 0.01c	0.20 ± 0.01a	0.20 ± 0.01a	2.13 ± 0.04b	0.50 ± 0.20b
*T. viride* + *P. fluorescens* + K_2_SiO_3_	48.86 ± 0.34a	5.55 ± 0.32c	44.02 ± 0.49b	0.62 ± 0.03a	4.64 ± 0.07a	0.06 ± 0.01d	0.13 ± 0.0b	0.18 ± 0.01ab	2.66 ± 0.06b	0.26 ± 0.01b
*E. camaldulensis* LE + K_2_SiO_3_	49.79 ± 0.54a	4.32 ± 0.33c	43.80 ± 0.48b	0.51 ± 0.03a	2.23 ± 0.05b	0.05 ± 0.01d	0.14 ± 0.01b	0.20 ± 0.03a	4.55 ± 0.06 a	0.69 ± 0.03 b
*C. sinensis* LE + K_2_SiO_3_	43.54 ± 0.28b	1.10 ± 0.20d	47.47 ± 0.39a	0.24 ± 0.02c	0.87 ± 0.03c	0.10 ± 0.01c	0.12 ± 0.01b	0.16 ± 0.01ab	3.15 ± 0.04ab	0.14 ± 0.01b
*F. benghalensis* FE + K_2_SiO_3_	41.90 ± 0.33b	9.89 ± 0.41b	41.99 ± 0.52c	0.40 ± 0.03b	5.64 ± 0.08a	0.12 ± 0.02c	0.14 ± 0.01b	0.11 ± 0.01 b	3.44 ± 0.06ab	0.72 ± 0.031b

*Letters in figure indicated that means ± S.E of treatments with the same letter/s were not significantly different according to Tukey's HSD at 0.05 level of probability*.

### DPPH Scavenging Activity

**Figure 4:**
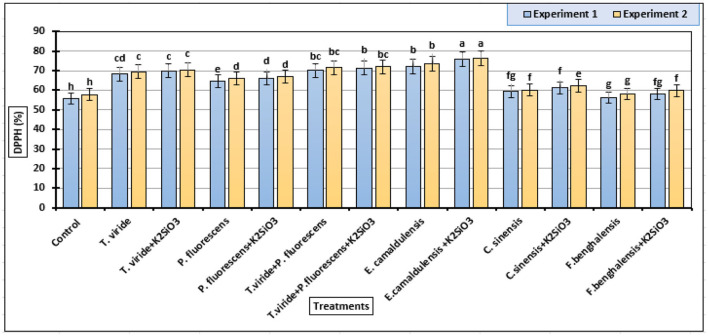
DPPH (2,2-diphenyl-1-picrylhydrazyl) inhibition percentages (means ± S.E) of methanol extracts from zucchini fruits as affected by the plant extracts, microbial inoculations, and potassium silicate biostimulants. Letters in the figure indicated that means ± S.E of treatments with the same letter/s were not significantly different according to Tukey's HSD at a 0.05 level of probability.

In Figure 4, all DPPH scavenging activity data of the zucchini fruits parameter were significantly influenced by the biostimulant applications. Our data indicated that the *E. camaldulensis* LE + K_2_SiO_3_ treatment increased the DPPH scavenging activity mean value of zucchini fruits (75.93 and 76.21%) followed by *E. camaldulensis* LE (72.24 and 73.45%) in the first and second experiments, respectively (Figure 4). In addition, the combination of K_2_SiO_3_ with plant extract and microbial stimulants had no effect on improving DPPH in zucchini fruits, in both experiments.

### Total Phenolic and Flavonoid Contents

**Figure 5:**
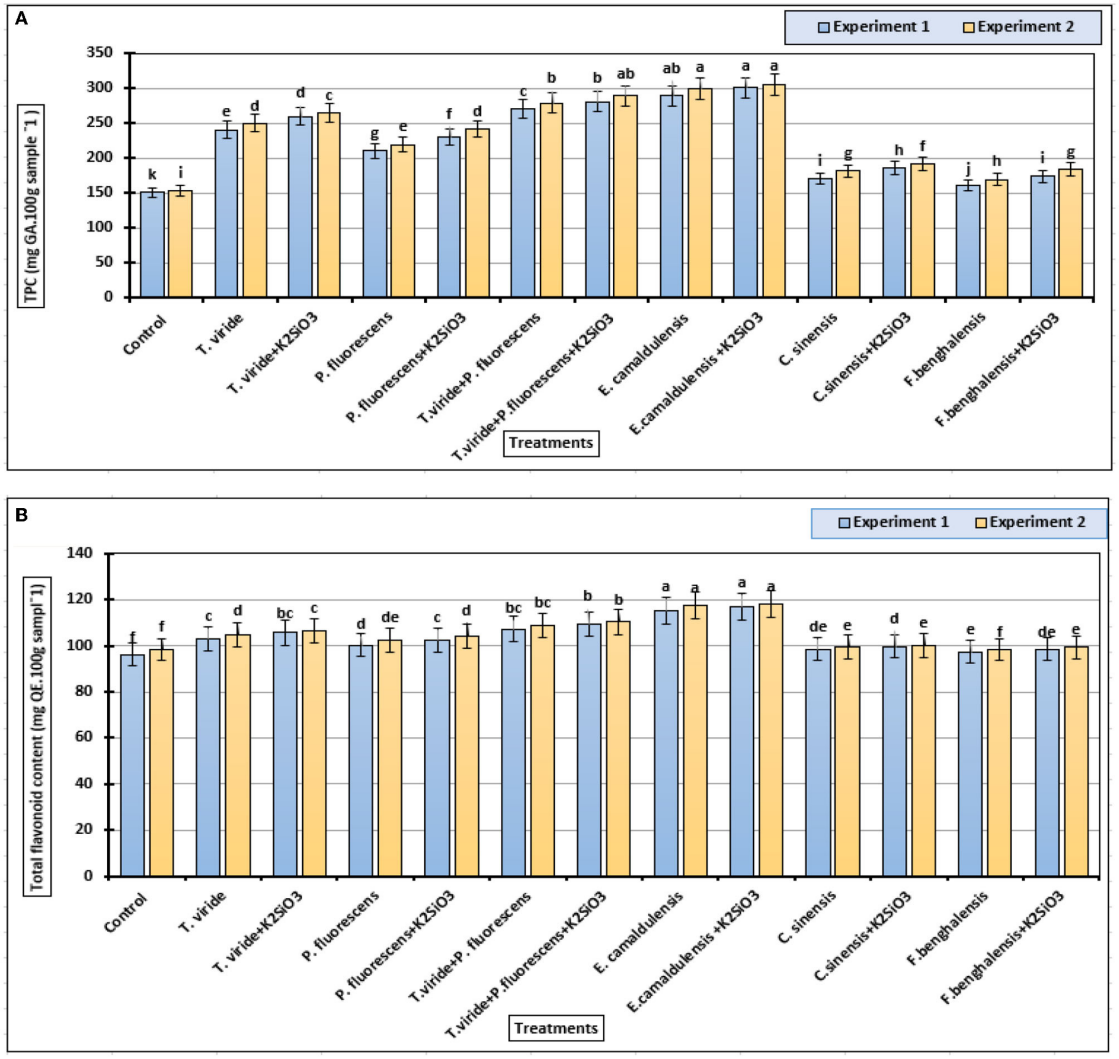
Phenolic and flavonoid contents (means ± S.E) of zucchini fruits as affected by the plant extracts, microbial inoculations, and potassium silicate biostimulants. **(A)** Total phenolic content and **(B)** total flavonoid content. Letters in the figure indicated that the means ± S.E of treatments with the same letter/s were not significantly different according to Tukey's HSD at a 0.05 level of probability.

Significant differences were found among the total phenolic contents of zucchini fruit samples in (Figure 5A), in both experiments. The values varied in a wide range with an average value of 150 to 300 mg GAE/100 g FW. In our two experimental conditions, the highest value was found with the treatment *E. camaldulensis* LE + K_2_SiO_3_ (300 mg GAE/100 g FW), followed by *E. camaldulensis* LE (289 mg GAE/100 g FW) and *T. viride* + *P. fluorescens* + K_2_SiO_3_ (287 mg GAE/100 g FW), whereas the lowest content was found in the control treatment.

The higher values of flavonoid content were recorded in biostimulant-treated zucchini plants with *T. viride* + *P. fluorescens* (104 mg QE/100 g sample), *T. viride* + *P. fluorescens* + K_2_SiO_3_ (110 mg QE/100 g sample), *E. camaldulensis* LE (116 mg QE/100 g sample), and *E. camaldulensis* LE + K_2_SiO_3_ (115 mg QE/100 g FW sample) treatments, with no significant difference among other treatments (Figure 5B). On the other hand, the spraying with K_2_SiO_3_ did not show any effects on the total flavonoids' content of zucchini fruits in both experiments.

### HPLC Analysis of Phenolic Compounds in Zucchini Fruits

Table 4 presents the phytochemicals in terms of the phenolic compounds identified in the methanol extracts (MEs) of zucchini fruits treated with plant extracts and microbial with K_2_SiO_3_ added only, which are six treatments in addition to the control treatment. The HPLC separation chromatograms are shown in Supplementary Figure S1. Ferulic (21.12 μg/ml), caffeic (19.63 μg/ml), and ellagic (18.33 μg/ml) acids were the most abundant compounds in zucchini fruit ME as affected by control. Zucchini fruit ME from plants treated with *T. viride* + K_2_SiO_3_ showed the presence of eugenol (35.16 μg/ml) and ellagic acid (17.36 μg/ml) as the main compounds. Eugenol (18.05 μg/ml) and caffeic acid (16.26 μg/ml) were the abundant compounds in ME from zucchini fruit treated with *P. fluorescens* + K_2_SiO_3_. Interestingly, α-tocopherol (22.01 μg/ml) with syringic acid (13.30 μg/ml) were the most abundant compounds in ME from fruits treated with *T. viride* + *P. fluorescens* + K_2_SiO_3_. The ME from fruits of the plants treated with *E. camaldulensis* LE + K_2_SiO_3_ identified *p*-coumaric acid (25.51 μg/ml), ferulic acid (20.11 μg/ml), and caffeic acid (18.87 μg/ml) as the abundant compounds. The ME of fruits from plants treated with *C. sinensis* LE + K_2_SiO_3_ identified pyrogallol (28.5 μg/ml), ferulic acid (18.09 μg/ml), and gallic acid (12.66 μg/ml) as the abundant compounds. Syringic (18.69 μg/ml) and caffeic (15.26 μg/ml) acids were the most abundant compounds in ME from fruits of zucchini collected from plants treated with *F. benghalensis* FE + K_2_SiO_3_.

**Table 4:** Effect of plant extracts, microbial inoculations, and potassium silicate biostimulants on phenolic compounds identified in zucchini fruits methanol extract in the second experiment.

**Compound**	**Phenolic compounds (μg/ml) in zucchini fruits ME**						
	**as plants treated with**						
	**Control**	***T.viride*** **+ K_**2**_SiO_**3**_**	***P. fluorescens*** **+ K_**2**_SiO_**3**_**	***T. viride* + *P. fluorescens*** **+ K_**2**_SiO_**3**_**	***E. camaldulensis*** **LE + K_**2**_SiO_**3**_**	***C. sinensis*** **LE + K_**2**_SiO_**3**_**	***F. benghalensis*** **FE + K_**2**_SiO_**3**_**
Myricetin	nd	10.33	nd	nd	nd	nd	nd
Syringic acid	9.22	9.14	8.12	13.30	nd	nd	18.69
*p*-Coumaric acid	9.68	nd	nd	8.09	25.51	8.23	nd
Eugenol	nd	35.16	18.05	nd	nd	nd	4.36
Vanillin	nd	nd	nd	nd	5.42	7.55	nd
Caffeic acid	19.63	6.47	16.26	5.36	18.87	6.98	15.26
4-Hydroxybenzoic acid	nd	nd	nd	nd	nd	7.12	nd
Pyrogallol	14.51	nd	nd	nd	nd	28.5	3.75
Gallic acid	nd	8.16	7.14	nd	5.12	12.66	12.44
Ascorbic acid	nd	nd	nd	nd	10.61	8.23	nd
Ferulic acid	21.12	nd	nd	6.12	20.11	18.09	nd
α-Tocopherol	7.45	nd	nd	22.01	nd	nd	nd
Salicylic acid	nd	9.12	9.56	nd	nd	nd	nd
Catechol	5.18	nd	nd	6.23	nd	nd	nd
Ellagic acid	18.33	17.36	8.49	5.14	nd	nd	nd
Protocatechuic acid	nd	10.68	2.21	nd	nd	nd	nd

*nd, not detected; ME, methanol extract*.

## Discussion

The development of eco-friendly products to improve the growth and yield of horticulture has spurred a massive interest in commercial, especially in poor countries. The use of biostimulants has become increasingly common in modern agriculture and in the global market for the sale of agricultural products (Xu and Geelen, 2018). The biostimulants used may be a substance of natural origin or microorganisms that work or a mixture of them that improve the condition of crops and resist some pathogens and stress conditions to which plants are exposed without causing harmful side effects to the environment or humans (Du Jardin, 2015). The advantage of using biomass contained in plant extracts is its low cost (Tembo et al., 2018). Conversion of plant biomass in plant extracts, showing the action of biostimulants or plant growth, can be supportive to farmers in developing countries that cannot afford synthetic biostimulants, because of their high costs (Fite et al., 2020).

In this study, photosynthetic pigments and productivity were affected by the application of different biostimulants used. The results illustrated generally that *E camaldulensis* LE or *Trichoderma viride* with K_2_SiO_3_ increased the previous characters. *Eucalyptus* leaf extract increased photosynthetic pigments and the production of zucchini plants. These results might be due to many species of eucalyptus have high rich in carotenoids, carbohydrates, phenols, flavonoids, and antioxidants. Due to these compounds detected in *E camaldulensis* LE, it can be considered a biostimulant to enhance growth and productivity. Also, allelopathic activity and this activity can be the important catalysts in reducing diseases and increasing total yield (El-Rokiek et al., 2019). The induced metabolism of the photosynthetic pigments signified the increased growth parameters of the used leaf extract. In this response, the photosynthetic pigments of seedling broccoli significantly increased by *Eucalyptus* leaf extract (Mohsen et al., 2018). Furthermore, the application of *T. viride* seems to have affected photosynthetic pigments and the total yield characteristics of *Brassica* leafy crops similar to what has been shown for the control. *Trichoderma* is a genus of filamentous fungi that include several species described as biostimulants and/or biological control agents in agriculture (Velasco et al., 2021). Almost, microbial is widely used as a biofertilizer almost for all crops with or without amendments (Vinale et al., 2008; Schuster and Schmoll, 2010; Reynolds, 2016). Similarly, the PGPR increases both the growth parameters and yield attributes of onions and zucchini with the triple inoculation treatments (Tinna et al., 2020; Novello et al., 2021).

The role of microbial or *Trichoderma* in increased crop yield and quality was achieved mainly by the ability to degrade complex organic compounds present in the soil. Complex organic compounds were made available to plants in a simpler form, so that they could be absorbed (Khan et al., 2017; Thapa et al., 2020).

It is clear from previous studies that treating plants with silicon benefits their leaf structure by improving plant leaf erection, which leads to increased light interception and reduced self-shading, which leads to improved photosynthesis (Galindo et al., 2021). In this regard, silicon has a positive effect on chlorophyll pigments, which leads to an improvement in growth, which in turn affects the production of plants (Thorne et al., 2020; Salim et al., 2021). It is noted that there is an effect of the use of biostimulants on the metabolism of plants and the final quality (Colla et al., 2015). Plant growth, quality, tolerance to abiotic and biotic stresses, photosynthesis, and using nutrients, fertilizers, and water are able to be enhanced by plant-derived biostimulants (PDBs) by modulating plant biochemical, molecular, and physiological processes (Rouphael and Colla, 2020b; Godlewska et al., 2021; Mosa et al., 2021b). Our previous work (Hassan et al., 2021) showed that the most abundant phenolic compounds in *Eucalyptus camaldulensis* LE were pyrogallol, caffeic acid, and *p*-coumaric acid, in *Citrus sinensis* LE were syringic and ferulic acids, and in *Ficus benghalensis* were gallic, p-coumaric, and syringic acids.

In addition, the application of biostimulants increased the biochemical compounds with potential effect on the nutrient content of plants. The shorter growing cycle of plants may result in biostimulation if the beneficial microbe has higher performance (i.e., *Trichoderma viride*), in improving the N uptake efficiency (Fiorentino et al., 2018). Moreover, the increase in the root system caused by the application of *Trichoderma* to the roots may have contributed to the increased N uptake of zucchini plants. This has also been observed when correlating the use of *Trichoderma* with the N content of leaves from several vegetable crops such as lettuce and tomato (Fiorentino et al., 2016; Sani et al., 2020).

Similar observations to our results were found after the use of microbial based on the enhanced yield by microbial inoculants has been linked in some cases to increased nutrient uptake and improved nutrient status of the cucumber and lettuce (Abdelaziz and Pokluda, 2009; El-Saady and Omar, 2017). Furthermore, some reports showed that *Pseudomonas* spp. was significantly increased the uptake of P, Fe, Ca, and manganese (Mn) in some vegetables, e.g., eggplant and tomato (Calvo et al., 2014; Chrysargyris et al., 2020).

Some studies that are similar to our results were found after the use of PDBs in agriculture leads to an improvement in the crop's quality with an increase in bioactive components (Di Mola et al., 2019). It can be said that biological stimulants have a positive effect on a number of chemical properties of fruits and vegetables. It is evident that the use of several types of PDBs has potential effects on the development and quality of several plant species, for example, tomato (Souri and Bakhtiarizade, 2019), rocket (Di Mola et al., 2019), and zucchini (Hassan et al., 2021).

Consistence with the present findings, PDBs as the foliar application were enhanced the total phenolic contents, antioxidant activity, and nutrient contents of tomato plants compared to the control (Chiaiese et al., 2018). Furthermore, these findings, generally, agreed with those previously reported on spinach and broccoli (Fan et al., 2011; Kałuzewicz et al., 2017), where they concluded that the use of biostimulants enhanced the phenolic content.

Foliar application of Si has biostimulation effects, and also, it can be used by plants to augment their defenses against the entrance of toxic ions *via* the root apoplast (Verma et al., 2021b).

The phenolic contents were ranged between 96.2 and 117.3 mg GAE/100 g of FW, as affected by different biostimulant treatments. Other studies showed that the average phenolic content of zucchini fruits of was 8.67 mg GAE/g FW (Hamissou et al., 2013).

Zucchini plants treated with the following treatments *T. viride* + K_2_SiO_3_, *P. fluorescens* + K_2_SiO_3_, *T. viride* + *P. fluorescens* + K_2_SiO_3_, *E. camaldulensis* LE + K_2_SiO_3_, *C. sinensis* LE+K_2_SiO_3_, and *F. benghalensis* FE + K_2_SiO_3_ showed several phenolic compounds (myricetin, syringic acid, *p*-coumaric acid, eugenol, vanillin, caffeic acid, 4-hydroxybenzoic acid, pyrogallol, gallic acid, ascorbic acid, ferulic acid, α-tocopherol, salicylic acid, catechol, ellagic acid, and protocatechuic acid) in the methanol extract with different concentrations compared with the control treatment. These phenolic compounds or PDBs are playing the important roles in regulating plant metabolic processes (Boudet, 2007; Lin et al., 2016; Mosa et al., 2021a). Simple phenolic acids such as hydroxybenzoic and hydroxycinnamic acids are derived from phenylpropanoid pathway (Mandal et al., 2010).

This study clarified the importance of the role played by Si element as a biostimulant in spraying on zucchini plants and increasing the efficiency of plant extract or microbial biostimulant. Si improves the growth of the plant by role defending the plant by increasing some different biochemical mechanisms in plants such as antimicrobial enzymes, flavonoids, and pathogen-related proteins (Verma et al., 2021a), chlorophyll pigments, and antioxidants (Thorne et al., 2020; Salim et al., 2021). Studies have also shown that one of the beneficial properties of Si is its positive effects on abiotic stress tolerance and resistance to pathogens and diseases and thus has an effective role in improving overall productivity (Savvas and Ntatsi, 2015; Abd-Alkarim et al., 2017; Shwethakumari et al., 2021).

Plant-derived biostimulants have a positive effect on crop and vegetable plants; however, to improve their efficacy and optimize their inclusion in the industrial processes, the understanding of their action mechanism should be amended (Brown and Saa, 2015; Backer et al., 2018; Rouphael and Colla, 2020a).

## Conclusion

The results of this study highlight the importance of biostimulant application to mitigate the effects of yield and biochemical. The potential use of some leaf extracts or microbial with K_2_SiO_3_ as a biostimulant was tested in zucchini plants on plant photosynthetic pigments, productivity, bioactive componence, and elements content parameters that were evaluated after its use. The productivity of zucchini plants was increased by the foliar application of the treatments *T. viride* + K_2_SiO_3_ or *E. camaldulensis* LE + K_2_SiO_3_ as well as the content of the leaves of photosynthesis pigments. HPLC analysis of phenolic compounds in zucchini fruits and elements content of leaves was affected by biostimulants (microbial and plant extract with silicon). Total phenolic contents and DPPH scavenging activity were increased significantly in plants treated with *E. camaldulensis* LE + K_2_SiO_3_.

## Data Availability Statement

The original contributions presented in the study are included in the article/Supplementary Materials, further inquiries can be directed to the corresponding author/s.

## Author Contributions

HH, AM, MS, and DA-E: conceptualization, formal analysis, and data curation. HH, AM, MS, AA-H, and DA-E: methodology. HH, AM, MF, MS, HA, and DA-E: software, investigation, and visualization. HH, AM, MF, AA-H, and DA-E: validation. HH, AM, MF, MS, AA-H, HA, and DA-E: resources and writing—original draft preparation. HH, AM, MF, MS, HA, AA-H, and DA-E: writing, reviewing, and editing. MF and MS: supervision. DA-E: project administration. HA and AA-H: funding acquisition. All authors have read and agreed to the published version of the manuscript.

## Funding

This research was funded by the Deanship of Scientific Research, King Saud University, through the Vice Deanship of Scientific Research Chairs.

## Conflict of Interest

The authors declare that the research was conducted in the absence of any commercial or financial relationships that could be construed as a potential conflict of interest.

## Publisher's Note

All claims expressed in this article are solely those of the authors and do not necessarily represent those of their affiliated organizations, or those of the publisher, the editors and the reviewers. Any product that may be evaluated in this article, or claim that may be made by its manufacturer, is not guaranteed or endorsed by the publisher.
