# Supplementary material for: Photosynthetic Pigments and Biochemical Response of Zucchini (Cucurbita pepo L.) to Plant-Derived Extracts, Microbial, and Potassium Silicate as Biostimulants Under Greenhouse Conditions
Source: Front Plant Sci. 2022 May 18;13:879545. doi: 10.3389/fpls.2022.879545 (PMC9159351; doi:10.3389/fpls.2022.879545)
Supplement: Supplementary file 1 [file Data_Sheet_1.docx]

**Photosynthesis pigment and** **Biochemical Response of Zucchini (*Cucurbita pepo* L.) to plant-derived extracts, microbial and potassium silicate as biostimulants under Greenhouse conditions**

Running title: Zucchini plants with promoting biostimulants

**Doaa Y. Abd-Elkader ^1^**, **Abeer A. Mohamed ^2^, Mostafa N. Feleafel ^1^, Mohamed Z. M. Salem ^3,*^, Asma A. Al-Huqail ^4^, Hayssam M. Ali ^4,**^, and Hanaa S. Hassan ^1^**

^1^ Department of Vegetable, Faculty of Agriculture (EL-Shatby), Alexandria University, Alexandria 21545, Egypt; doaa.abdelkader@alexu.edu.eg (D.Y.A.-E.); hanaa.saad@alexu.edu.eg (H.S.H.); mostafa.feleafel@alexu.edu.eg (M.N.F.);

^2^ Plant Pathology Institute, Agriculture Research Center (ARC), Alexandria 21616, Egypt; abeer_pcr@yahoo.com

^3^ Forestry and Wood Technology Department, Faculty of Agriculture (El-Shatby), Alexandria University, Alexandria 21545, Egypt; zidan_forest@yahoo.com

^4^ Chair of Climate Change, Environmental Development and Vegetation Cover, Department of Botany and Microbiology, College of Science, King Saud University, Riyadh 11451, Saudi Arabia;; [aalhuquail@ksu.edu.sa](mailto:aalhuquail@ksu.edu.sa) (A.A.A.-H.); hayhassan@ksu.edu.sa (H.M.A.)

^5^

***** Correspondence: [zidan_forest@yahoo.com](mailto:zidan_forest@yahoo.com) (M.Z.M.S.); hayhassan@ksu.edu.sa (H.M.A.)

Table S1. Irrigation scheduling during different growth stages of zucchini, coefficients (Kc), evapotranspiration (ET0) and water requirements (ETc), under the greenhouse, in both experiments.

| Growth stages | First experiment | | | | Second experiment | | | |
| --- | --- | --- | --- | --- | --- | --- | --- | --- |
|  | Establishment | Vegetative | Flowering | Fruits formation | Establishment | Vegetative | Flowering | Fruits formation |
| Number of days per stage | 15 | 20 | 40 | 15 | 15 | 15 | 30 | 10 |
| Crop Coefficients (K_C_) | 0.41 | 0.70 | 0.95 | 0.90 | 0.41 | 0.70 | 0.95 | 0.90 |
| Evapotranspiration (ET_0_) mm day^-1^ inside the greenhouse =73% from outside the greenhouse | 4.5 | 3.2 | 2.7 | 1.7 | 1.8 | 1.9 | 2.3 | 2.6 |
| Water requirements for zucchini crop (ET_c_) (mm day^-1^) | 1.85 | 2.24 | 2.57 | 1.53 | 0.74 | 1.33 | 2.19 | 2.34 |
| Total water requirements per growth stage (mm) | 27.7 | 44.8 | 102.6 | 22.95 | 11.07 | 19.95 | 65.55 | 23.40 |


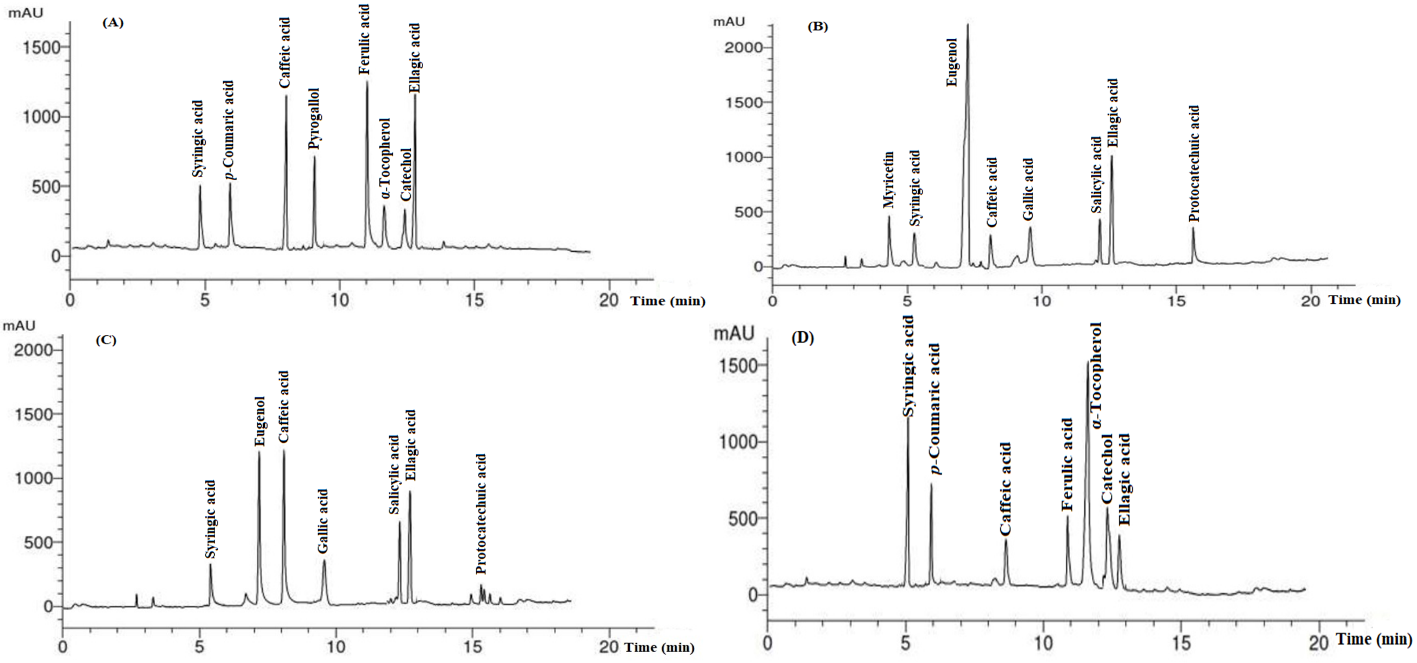


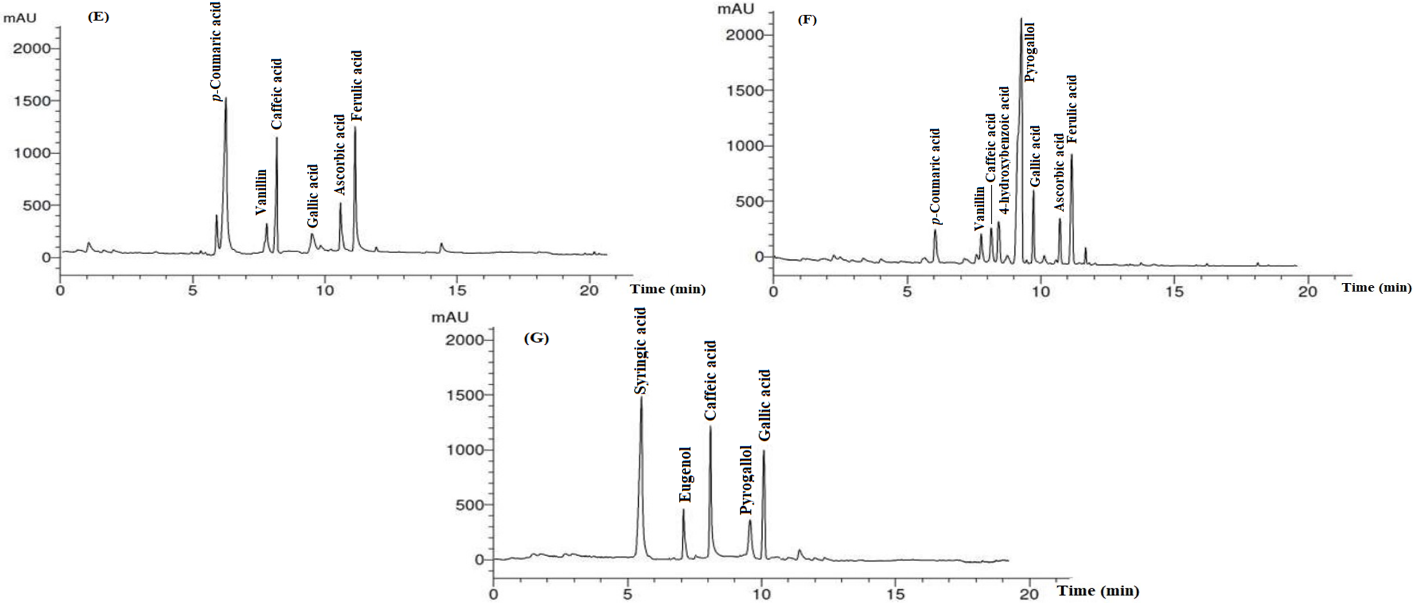


**Figure S1.** Effect of plant extracts, microbial inoculations, and potassium silicate biostimulants on HPLC chromatograms of phenolic compounds identified in methanol extracts from zucchini fruits. (A) Control; (B) *T. viride*+ K_2_SiO_3_; (C) *P. fluorescens*+K_2_SiO_3_; (D) *T. viride*+*P. fluorescens*+K_2_SiO_3_; (E) *E. camaldulensis* LE+K_2_SiO_3_; (F) *C. sinensis* LE+K_2_SiO_3_; (G) *F. benghalensis* FE+K_2_SiO_3_
